# ‘Put in a room and left’: a qualitative study exploring the lived experiences of COVID-19 isolation and quarantine among rehabilitation inpatients

**DOI:** 10.1080/07853890.2022.2155698

**Published:** 2022-12-20

**Authors:** Subbuh Luker, Kate Laver, Rachel Lane, Elizabeth Potter, AnnMarie Harrod, Petra Bierer, Zoe Adey-Wakeling, Jonathan Karnon, Ian D. Cameron, Maria Crotty

**Affiliations:** a Division of Rehabilitation, Aged and Palliative Care, Southern Adelaide Local Health Network, Flinders Medical Centre, Adelaide, Australia; b College of Medicine and Public Health, Flinders University, Adelaide, Australia; c John Walsh Centre for Rehabilitation Research, Northern Sydney Local Health District and Faculty of Medicine and Health, University of Sydney, Sydney, Australia

**Keywords:** Rehabilitation, COVID-19, isolation, quarantine, experience

## Abstract

**Background:**

The SARS-CoV-2 (COVID-19) pandemic has resulted in significant changes to health care delivery and the patient’s experience in hospital. Changes for those who contracted COVID-19 or were close contacts included isolation and quarantine, visitor restrictions and changes to usual models of care to reduce viral transmission. Traditional models of inpatient rehabilitation utilise communal spaces (e.g. shared gyms and dining rooms) and involve a multidisciplinary team interacting with the patient daily.

**Objectives:**

To report the experience of COVID-19 related isolation and quarantine among rehabilitation inpatients and their family members who experienced a nosocomial outbreak and to make recommendations for rehabilitation units.

**Methods:**

A qualitative phenomenological methodology using semi-structured telephone interviews.

**Results:**

19 semi-structured interviews were conducted comprising of 13 general rehabilitation inpatients and 6 family members. Five themes were established: (1) the impact of social and physical isolation; (2) boredom and limited access to therapy; (3) the impact of technology; (4) inadequate information sharing and (5) positive experiences and things done well. Several novel insights were identified including: the desire for increased social interaction from staff to compensate for a lack of visitors; the impact of physical and cognitive deficits on a patient’s ability to reach basic items around their room or call for help, and the unique impact of isolation and quarantine on individuals who have a history of trauma, discrimination or mental illness.

**Conclusions:**

This study establishes key areas that should be considered by rehabilitation units globally to adjust traditional models which are not suitable in this pandemic. Strategies to mitigate the impact of isolation include providing training to increase use of technologies such as tablet devices, increased staff social engagement to reduce isolation and tailoring the environment to suit specific patient groups.

**KEY MESSAGES**
COVID-19 related isolation and quarantine has a significant and unique impact on patients with cognitive and physical impairments such as those in inpatient rehabilitation. Patients who are required to isolate in inpatient settings expressed a desire for compensatory increased social interaction from staff and required specific assistance with basic daily tasks while isolated. The study makes key recommendations for other rehabilitation units to integrate into their approach for managing patients required to isolate or quarantine.

## Introduction

Since the first reported cases of SARS-CoV-2 (COVID-19) in December 2019, the impact on health systems around the world has been devastating. To date, there have been over 539 million cases of COVID-19 and over 6 million deaths worldwide [1]. Authorities have introduced strict public health measures to prevent transmission including social distancing, isolation, quarantine requirements, mask use and mass testing by health authorities as well as self-testing using rapid antigen kits [2–5]. Hospitals and health care facilities have been identified as high-risk sites for transmission due to the large numbers of people entering and leaving, immunocompromised patients, patient facing roles and close personal exposure between COVID-19 patients and staff [6]. Consequently, these sites have introduced restrictions to reduce virus transmission to vulnerable patients and vital health care workers. These requirements have fluctuated over time but have included: isolation of patients with COVID-19, quarantining of ‘close contacts’ and restricting visitors. Numerous studies, predating the COVID-19 pandemic, have shown that isolation in general is associated with cognitive decline [7], low mood [8] and increased mortality [9–17]. Additionally, studies exploring the impact of isolation in hospital settings specifically have identified that patients experience a reduced sense of control [18,19], feel confined, abandoned or stigmatised [18–21] and perceive that they are receiving lower quality care [22]. In contrast, studies that have evaluated hospital visitation policies [23,24] have reported flexible visitation policies are associated with faster recovery times, reduced length of stay, lower staff burden, reduced patient and family stress and increased patient satisfaction [25–30].

To date, numerous studies have assessed the impact of the COVID-19 pandemic on a variety of outcomes including the rates of mortality, morbidity and hospitalisation, the impact on mental health and the impact on service delivery across various acute settings. However, to our knowledge, no studies have assessed the impact of COVID-19 related isolation and quarantine in a rehabilitation setting. This is a unique environment and cohort of patients that are admitted for therapy which requires them to work closely with many people and move about, all of which are difficult to achieve within COVID-19 isolation and quarantine requirements.

Inpatient rehabilitation is traditionally undertaken in a hospital environment where patients work with a large multidisciplinary team to maximise function and independence in daily tasks, improve psychological wellbeing, and engage in social activities after an illness or impairment [31]. By design, these units often have several communal spaces for patients to interact with one another and with staff such as shared dining rooms, therapy rooms and gyms. It is through intensive daily therapy, social interaction and practice, that patients can regain or maximise function in order to discharge home. As a result of COVID-19 related changes to hospital policies, inpatient rehabilitation, like many other services has been affected. Previous studies have demonstrated isolation limits a patient’s ability to take part in rehabilitation activities [32]. Rehabilitation patients are often physically dependent, cognitively impaired, older and have had a prolonged hospitalisation, resulting in high nursing and therapy needs and hence caring for them while in isolation or quarantine poses a unique challenge. Whilst a previous study has demonstrated technology such as smart phones, videocalls, tablets devices and computers can assist to alleviate the impact of isolation in a general inpatient setting [33], the use and acceptability of technology for older patients and those with cognitive and physical impairments in an inpatient rehabilitation setting has not been specifically investigated.

The aim of this study was to understand the experience of COVID-19 related isolation and quarantine among rehabilitation inpatients and their family members involved in a nosocomial outbreak and to describe the role technology played in mitigating isolation. Based on these findings, the aim was to suggest practice changes for rehabilitation units, which could improve patient experiences of nosocomial outbreaks when transfer off the ward does not occur.

## Methods

### Design

The study used a qualitative phenomenological design through interviews with affected participants and their family members and subsequent thematic analysis using an inductive approach. Interviews were analysed using Braun and Clarke’s Six Step Framework for Thematic Analysis [34] which include [1] familiarisation with the data [2], generation of initial codes [3], theme search [4], theme review [5], definition and naming of themes and [6] production of the report [34]. This research was reported in accordance with the items in the Consolidated Criteria for Reporting Qualitative Studies (COREQ) 32 item checklist [35].

### Participants

Ethics review exemption was received by the Southern Adelaide Local Health Network Human Resources Ethics Committee under the ‘low risk, quality improvement’ pathway. Eligible participants were a) patients who had been required to isolate for fourteen days (COVID-19 positive cases) or quarantine for ten days (close contacts) in an inpatient rehabilitation unit within the Southern Adelaide Local Health Network or b) family members of such patients. Family members were either relatives of interviewed patients (*n* = 3) or relatives of patients who were required to isolate or quarantine during the study period but were unable to be interviewed directly (*n* = 3). Patients who were in isolation or quarantine were cared for in a private room with an ensuite, window, television, corded telephone, chair and tray table. Patients were offered tablet devices when available to assist with contacting family and alleviating the impact of isolation. Eligible participants were identified by their rehabilitation team and invited to participate by a staff member. Invited participants were provided with a written information sheet outlining the study aims. If agreeable they provided both verbal and signed written consent and were interviewed by a trained research assistant. Participants were recruited and interviewed between the 1st of January 2022 and the 2nd of March 2022 until data saturation occurred.

### Data collection

Telephone interviews were conducted by a research assistant. Patient and family members were interviewed separately. At the time of interview, patients were either in isolation or quarantine on a rehabilitation ward, had completed their isolation or quarantine but remained in the rehabilitation ward or had been discharged home. Interviews were conducted using a semi-structured interview guide (see Appendix A). Researchers were able to seek clarification and encourage participants to expand on their responses to enable greater understanding. Interviews were audio recorded and transcribed verbatim by a professional transcription company. Data was uploaded into QSR NVivo [36] for analysis. Data were analysed using an inductive approach following Braun and Clarke’s Six Step Framework for Thematic Analysis whereby two researchers (S.L. and R.L.) separately: read each interview and [1] familiarised themselves with the data [2], generated initial codes [3], searched for themes and [4] reviewed the themes [34]. A third researcher (K.L.) analysed 25% (5 of 19 interviews) following the aforementioned steps 1–4 to validate the results. Following this, all three researchers met and discussed their findings and in collaboration [5]: defined and named the overarching themes and [6] produced the report.

## Results

### Participant demographics

A total of 19 participants were recruited to the study comprising of patients who were required to quarantine or isolate (*n* = 13) and family members of these patients (*n* = 6). Most patients were quarantined as ‘close contacts’ exposed to the virus (12/13) and all but one patient had completed their isolation or quarantine at the time of interview. The mean (SD, median) age of patients was 71 (19.3, 74) years. The majority (9/13) were female. One patient identified as Aboriginal or Torres Strait Islander. In patients who had completed isolation or quarantine the mean (SD, median) duration from exit day of isolation or quarantine to interview was 6.7 (4.2, 5) days. The mean (SD, median) duration from exit day of isolation or quarantine to interview of a family member was 8.2 (6.1, 5) days. Further patient demographics are outlined in Table 1.

**Table 1.** Patient demographics.^a^

ID	Age range	Sex	Reason for admission	COVID + or close contact	Active vs completed isolation or quarantine at time of interview	Family member interview
1	50–59	F	Orthopaedic rehabilitation	COVID positive	Completed	N/A
2^b^	80–89	F	Medical reconditioning	Close contact	Active	Son
3	70–79	F	Orthopaedic rehabilitation	Close contact	Completed	N/A
4	60–69	F	Surgical reconditioning	Close contact	Completed	N/A
5^b^	80–89	M	Ischaemic stroke	Close contact	Completed	Daughter
6^b^	80–89	F	Orthopaedic rehabilitation	Close contact	Completed	Daughter
7	90–99	F	Orthopaedic rehabilitation	Close contact	Completed	N/A
8	80–89	F	Medical reconditioning	Close contact	Completed	N/A
9	60–69	F	Medical reconditioning	Close contact	Completed	N/A
10	80–84	M	Orthopaedic rehabilitation	Close contact	Completed	N/A
11	60–69	F	Neurological rehabilitation	Close contact	Completed	N/A
12	20–29	M	Medical reconditioning	Close contact	Completed	N/A
13	40–49	M	Medical reconditioning	Close contact	Completed	N/A
Patient demographics where family members were interviewed (patient did not interview)						
14^c^	90–99	F	Medical reconditioning	Close contact	Completed	Daughter
15^c^	60–69	F	Neurological rehabilitation	Close contact	Completed	Sister
16^c^	60–69	M	Medical reconditioning	Close contact	Completed	Sister

^a^Total of 13 patients interviewed, 3 family members of patients who were also interviewed and 3 family members of patients who were not interviewed.

^b^Demographics of 3 patients who were both interviewed directly and had family members interviewed.

^c^Demographics of 3 patients who were not directly interviewed but whose family members were interviewed.

### Themes

The study identified five overarching themes: (1) the impact of social and physical isolation; (2) boredom and limited access to therapy; (3) the impact of technology on isolation; (4) inadequate information sharing and (5) positive experiences and things done well.

#### Theme 1. The impact of social and physical isolation

##### The impact of social isolation

Almost all patients reported feelings of social isolation, loneliness, and missing meaningful contact with others. All family members were concerned about the psychological impact of isolation on their loved ones.

While participants acknowledged the negative effects of isolation, many stated this could have been lessened by increased social contact from staff. Patients reported staff only entered their rooms and interacted when necessary (such as to provide medication or meals) and left quickly which exacerbated feelings of loneliness and isolation.

If they could spend time talking – I'm not saying spending hours, but even if they could sit down and spend half an hour with someone, you know talk about whatever… Some patients may want to talk about current politics, some might want to talk about family, or their partner, or their pet… – Patient 13

Those who came in, they were in and out as quick as possible…. They’d come in and drop your medicine off and that’d be it. So, there was no real contact with them either…you’re always watching the door, waiting for somebody to come in to talk to – Patient 3

I think they needed to spend extra time with the patients. They said they did try, they were trying that, but I don’t actually know whether that happened or not – Daughter of patient 14

A third of family members commented that being in isolation or quarantine was no different to being hospitalised in general, and the experience of patients in isolation or quarantine was akin to any patient in a healthcare facility with visitor restrictions.

It was horrendous. It wasn’t just once he was isolated, the whole lockdown and not allowing visitors just because of COVID in general is horrendous for an 84-year-old man with dementia who relies on his carer and family member to even give an accurate medical history…Really, being isolated didn’t make a whole lot of difference because the ward was already in lockdown. Visitors were already banned … Yeah. The point I’m making is it wasn’t very different to the situation that it was the entire month. – Daughter of patient 5

Some patients reported that isolation or quarantine did not severely impact them. These patients reported that they preferred solitude or felt isolation was similar to their usual circumstances and thus did not perceive a significant difference.

Look, at my age, I think we cope with isolation much better than other younger people…I didn’t feel at all really isolated. I mean, there’s television, a paper to read, magazines, puzzles to do, people popping in and out. So, there really, for me, wasn’t a sense of isolation. – Patient 8

Sometimes you don’t like people always popping in and out of a place. So I rather enjoyed myself on my own …I didn’t mind very much because I’m quite used to liking my own company. – Patient 6

##### The impact of physical isolation

Isolation led to limited staff assistance at the bedside and as a result, patients with physical impairments reported significant difficulty in reaching items such as the call bell, meals and the telephone. As patients were required to close their doors, they were also unable to easily call out for help.

I used to be too far away to grab the phone – Patient 6

Because she wasn’t allowed to move around without someone with her – they put her walker across the room so she couldn’t walk around, and they’d put her in the chair. And then she couldn’t reach the telephone. So that was a problem. – Daughter of patient 6

The effect of being alone in a confined space with the door closed generated feelings of captivity, claustrophobia, and punishment in some patients. Patients with specific cultural backgrounds such as the Aboriginal and Torres Strait Islander population who have a history of trauma and being segregated reported this to be a triggering environment.

Well, it feels as though you didn’t – as though you weren’t wanted, to put it in some way, that you weren’t wanted. You were put in a room and left. Nobody knows how hard that is to cope with, mentally. It’s so hard to realise that you can’t walk out that door any time you like…and of course, I'm Aboriginal background, so shutting that door, that was terrible to me. Even though I keep it closed to keep the noise out, I know I can go and open it. But not being able to open that door, that was terrible. – Patient 3

The doors being shut. That was pretty horrible…Yes, I’m claustrophobic – Patient 7

One patient observed how isolating patients who were delirious or cognitively impaired worsened their disorientation, agitation, and fear.

I certainly know that the disoriented man and the disoriented woman were just - she kept, almost accusing the nursing staff of having stolen her bag. I suggested that she call her husband, and she didn’t know what his number was, and it was - they were just in a mess, and they were in a situation that they weren’t comfortable with, and they were completely disoriented. – Patient 1

#### Theme 2. Boredom and limited access to therapy

##### Boredom

The majority of patients and two family members reported isolation resulted in feelings of boredom and inadequate mental stimulation.

It’s your life gone; you haven’t got a life. You’ve got to stay there and do nothing. And all you’ve got is TV, books, that’s it. – Patient 3

Well, we tried to find stuff to do, but there wasn’t very much to do as we were in our rooms, so. – Patient 12

##### Limited access to therapy

Many patients and family members noted the significant impact of reduced access to specialised therapy such as hydrotherapy and the gym during isolation had on their functional improvements and rehabilitation progress.

I couldn’t go to the gym, so I missed all that time in the gym. And have just sat on my bottom on a chair and bemoaned the fact that I could be getting better, and I haven’t got any better. – Patient 7

As far as the lockdown goes, yes, I was able to walk across my room on my frame with the physio or her assistant, but not having the group activities really knocked most of us around, I’d say…there was no access to the gym, which is a big deal for me. – Patient 11

#### Theme 3. The impact of technology on isolation

Most patients reported that they communicated with family by telephone. Several participants were offered tablet devices to provide entertainment and access to video calls, however oftentimes they did not know how to use the device and staff support was limited. The minority of patients who were able to effectively use a tablet device or computer reported it reduced boredom and was valuable. Amongst those who were less technologically adept, several reported they would have liked increased assistance to learn. However, some felt that the barriers to use (e.g. cognitive impairment and inexperience) were too significant to overcome. One family member felt videocalls could not alleviate the need for true human connection and another participant acknowledged that while technology can be beneficial, they simply preferred not to engage with it.

Well, naturally they suggest things and technology, which you can’t use because of your ability – Patient 2

Because mum’s not technical, she’d need someone to set it up. – Daughter of patient 6

Video call, especially my mother’s generation … video calls are very pale and distant comparison to seeing people three dimensionally. – Son of patient 2

A friend of mine, her husband’s mother lives in England, so they would Facetime every Sunday on a computer so that the grandkids got to know their grandmother. So I know some people who use it normally, but it’s not my cup of tea – Patient 1

I’ve got a laptop computer on my desk, and I play games. So, it keeps me sane. – Patient 9

#### Theme 4. Inadequate information sharing

Several family members stated they received limited updates about their loved ones in isolation. They acknowledged staff pressures and offered alternatives such as updates from nurses or patient liaisons regardless of whether things were stable or unchanged. This was particularly important for family members of patients with cognitive impairment who reported they couldn’t rely on the patient for accurate information. Those who reported receiving regular communication stated it was highly valuable and reassuring.

Well, I think there should have been a phone call every day made to a family member while they were in lockdown by a staff member, by the nurse or the doctor – Daughter of patient 14

Some patients similarly expressed a need for increased information about their condition, isolation updates and their overarching care and discharging plan.

I’m aware that the situation keeps changing, but there seems to be an information breakdown for the nursing staff and the doctors. Also, it was never very clear to me what was happening all the time. – Patient 1

#### Theme 5. Positive experiences and things done well

Many patients and family members identified factors which improved their experience in isolation. This included a private room with adequate space to mobilise, a room with a view and windows that could be opened, provision of a television and assistive technology, the ability to individually control the room temperature and positive staff interactions.

The window was open. So you could get a little bit of fresh air if you wanted it. And that did make a lot of difference, that you could open the windows. – Patient 6

I know she was quite impressed with the television. She loved the fact that she had a big television. – Daughter of patient 6

We are very well looked after, with kind staff who want to do the best by you and always thinking of ways that you might be occupied. – Patient 11

She said they were very caring staff there, so that was nice. – Sister of patient 15

## Discussion

Overall, participants had similar experiences in isolation and quarantine. There was occasional divergence such as those who were accustomed to isolation and did not perceive a significant change. Isolation was a negative experience that impacted patient’s mood and impeded their rehabilitation however, patients were able to identify some aspects that were managed well and helped them cope. In sum, this study has established five overarching themes relating to the experience of isolation and quarantine among rehabilitation inpatients: (1) the impact of social and physical isolation; (2) boredom and limited access to therapy; (3) the impact of technology on isolation; (4) inadequate information sharing and (5) positive experiences and things done well. These findings provide valuable information for other rehabilitation units facing similar challenges.

The findings of this study are consistent with previous literature finding that isolated patients (in any setting) experience loneliness, stress, and depression [18,37–43]. In keeping with other studies [33], patients were generally accepting of visitor restrictions however, we identified that patients were significantly impacted by limited social interaction and time spent with hospital staff. Although there were no restrictions in place for staff in terms of how long they could spend in a patient’s room, the patients reported short visits and transactional interactions. This aligns with previous quantitative research which reported healthcare staff spent less time with isolated versus non-isolated patients [44] and that staff were two times less likely to enter the room of a patient in isolation with overall contact being two fold less in isolated patients [44].

Furthermore, a unique aspect of the rehabilitation population is that all patients have some form of impairment or disability limiting their daily function and hence require increased carer assistance, a concern given the finding that isolated patients are less likely to receive staff contact [44]. Our patients reported issues reaching vital items such as the call bell to request assistance, meals and the telephone, or were unable to return to bed or reposition without assistance. This aligns with the findings of Stelfox et al. [22], who reported isolated patients were up to eight times more likely to experience falls, ulcers and fluid and electrolyte abnormalities – all adverse events that are even more likely in isolated patients with concurrent disability and limited mobility, such as the rehabilitation cohort. These subthemes highlight the potential benefits of increased staffing ratios and staff social interaction, positioning of important objects within reach and regular patient assistance checks as disabled patients have unique needs compared to those who are able and mobile. It was also evident that time should be spent with patients to elicit their individual preferences.

This study identified important considerations when it comes to patients with diverse cultural backgrounds such as the Aboriginal and Torres Strait Islander population who have experienced institutional segregation and trauma and in whom standard isolation and quarantine procedures are inappropriate. Additionally, literature highlights the Aboriginal and Torres Strait Islander people’s connection to family and the environment is central to their wellbeing and hence isolation can have profound impacts [45]. We suggest these patients are managed with an individual care plan with input from the relevant cultural liaison and mental health experts to advise how to minimise harm.

Inpatient rehabilitation is a time limited, and goal directed program in which intensive therapy is provided by trained specialists. This includes one on one, group, hydrotherapy, gym and technology-based therapies as well as communal meals and activities to increase independence and social engagement. Thus, the impact of isolation and quarantine was more dramatic in rehabilitation patients than in other hospital settings as patients were anticipating or had become familiar with this arrangement. Consequently, the inability to access the same level of therapy while in isolation or quarantine affected their ability to progress towards discharge. When the communal spaces in rehabilitation are no longer accessible there is the potential to use technologies such as tablet devices and videoconferencing to deliver therapy, maintain relationships and allow communication. Some patients were set up with equipment and self-directed programs in their rooms to maximise time when they did not have an individual therapist visiting them in personal protective equipment, however, this relied upon them being physically and cognitively capable of using the equipment and on training around equipment use. We found that only a limited number of this patient group were able to benefit from this approach.

In terms of usability and acceptability of technological solutions to isolation this study found that only those rehabilitation patients who had pre-existing familiarity with devices such as *iPads* and computers were able to benefit from them, as insufficient time was allocated to train patients who have no prior experience with the equipment. Importantly, those patients who were familiar with the technologies and able to use them found them helpful. Several patients expressed willingness to learn how to use this technology but required one on one assistance which was challenging due to staff shortages. This is in contrast to previous studies that found patients were able to successfully use technology to alleviate social isolation and boredom in hospital [33,37,46]. However, these studies included younger and technologically experienced patients outside of a rehabilitation setting.

The need for improved information sharing between health professionals, patients and family members was evident and in keeping with numerous previous studies exploring the healthcare experience both relating to isolation and in general [37,38,47].

Our findings suggest that when outbreaks occur strategies should be adopted to mitigate the impact of isolation and quarantine on patients who remain on rehabilitation wards including, increased staffing ratios for rehabilitation patients in isolation and quarantine to provide disability specific assistance and tailored in-room therapy; increased social engagement from onsite staff to alleviate isolation; provision of dedicated training and staff support to increase use of technologies such as tablet devices and individualised and culturally appropriate care for vulnerable patient groups. Our findings also support formal protocols and systems be implemented to prevent adverse events and provide a structured approach to care for this patient cohort. This should include individualised nursing and occupational therapist review and room set up so that it is tailored to individual patient needs while in isolation as well as regular timed patient welfare checks. Overall these approaches will provide support, alleviate the impact of isolation and utilise technology to improve isolation and deliver therapy as patients recover.

Limitations of this study include recruitment from one health network in Southern Adelaide, in South Australia comprising of three geographically distinct sites. Recruitment from other health networks, cities and countries would increase generalisability of results and allowed for sub-analysis of patient experiences with certain demographics, illnesses, or impairments. Interviews were conducted by telephone only; this may have reduced the benefits of face-to-face communication and ability to assess body language and social cues.

## Conclusions

While there are studies exploring the impact of COVID-19 across numerous health areas and studies exploring the rehabilitation of patients who have had COVID-19 this study is unique in its exploration of the experience COVID-19 isolation and quarantine itself on rehabilitation inpatients, a distinct patient population who have substantial dependency and clear goals that are impeded by isolation and quarantine. It establishes key themes and provides recommendations for the care of these patients in isolation across rehabilitation as well as other hospital settings around the world.

## Data Availability

The data that support the findings of this study are available on request from the corresponding author [SL]. The data are not publicly available due to [restrictions e.g. their containing information that could compromise the privacy of research participants].

## Appendix A. Interview guide for patient or family member

1. Are you (or is your family member) currently isolated? How long ago since you (or your family member) were de- isolated?
2. Can you describe what happened to you and why you were isolated?
3. What aspects of being isolated in your room were most difficult for you?
4. What could the hospital do to improve that experience for other patients?
5. Do you have suggestions for things that should be included in the room?
6. During your isolation did you use videoconferencing on an iPad to talk to your family?
7. Did you have any difficulty communicating with your family? How were you mainly doing it? Phone?
8. If you used and iPad did you have a technical issue with your iPad? Did you audio or video not work as it should?
9. Did you have any difficulty communicating with the doctors and nurses who were wearing gowns and masks?
10. What type of technology have you used in the past to access healthcare services or personal health information?

Question for family member only:

1. How would you rate your satisfaction with the communication you received?
2. What suggestions do you have for improving the communication process with other families?
